# Quantifying Microstructure Features for High-Performance Solid Oxide Cells

**DOI:** 10.3390/ma17112622

**Published:** 2024-05-29

**Authors:** Cristina Mariana Ruse, Lily Ann Hume, Yudong Wang, Thomas C. Pesacreta, Xiao-Dong Zhou

**Affiliations:** 1 Institute for Materials Research & Innovation, University of Louisiana at Lafayette, Lafayette, LA 70504, USA; yudong.wang@uconn.edu (Y.W.); thomas.pesacreta@louisiana.edu (T.C.P.);; 2 Department of Petroleum Engineering, University of Louisiana at Lafayette, Lafayette, LA 70504, USA; 3 Microscopy Center, University of Louisiana at Lafayette, Lafayette, LA 70504, USA; lily.hume@louisiana.edu; 4 Department of Chemical Engineering, University of Louisiana at Lafayette, Lafayette, LA 70504, USA

**Keywords:** solid oxide fuel cells (SOFCs), focused ion beam–scanning electron microscopy (FIB-SEM), microstructure, triple-phase boundary (TPB), 3D reconstruction

## Abstract

The drive for sustainable energy solutions has spurred interest in solid oxide fuel cells (SOFCs). This study investigates the impact of sintering temperature on SOFC anode microstructures using advanced 3D focused ion beam–scanning electron microscopy (FIB-SEM). The anode’s ceramic–metal composition significantly influences electrochemical performance, making optimization crucial. Comparing cells sintered at different temperatures reveals that a lower sintering temperature enhances yttria-stabilized zirconia (YSZ) and nickel distribution, volume, and particle size, along with the triple-phase boundary (TPB) interface. Three-dimensional reconstructions illustrate that the cell sintered at a lower temperature exhibits a well-defined pore network, leading to increased TPB density. Hydrogen flow simulations demonstrate comparable permeability for both cells. Electrochemical characterization confirms the superior performance of the cell sintered at the lower temperature, displaying higher power density and lower total cell resistance. This FIB-SEM methodology provides precise insights into the microstructure–performance relationship, eliminating the need for hypothetical structures and enhancing our understanding of SOFC behavior under different fabrication conditions.

## 1. Introduction

The necessity of reducing fossil fuel reliance while being mindful of the growing energy demand has led to an increasing interest in the design and development of solid oxide fuel cells (SOFCs). SOFCs are highly efficient and environmentally benign electrochemical devices capable of producing electricity using various fuels [1,2,3]. SOFCs represent an alternative to combustion processes and have twice the efficiency of internal combustion engines (>70% in combined heat and power systems). A generic SOFC is composed of a dense ceramic electrolyte sandwiched between two porous electrodes, and its microstructure has been studied and proven to be crucial in understanding cell performance [4,5,6,7,8]. Digital reconstruction gained attention in the early 2000s for better describing the reaction sites in the anode with the help of advanced focused ion beam (FIB)–scanning electron microscopy (SEM) [8,9,10,11,12,13,14] and X-ray nano-computed tomography (nano-CT) [15,16,17].

The microstructure of an SOFC anode, a ceramic–metal composite, is highly dependent upon the arrangement of the yttria-stabilized zirconia (YSZ) and nickel (Ni) phases [18,19]. The functional layer of the anode offers electrochemically active sites for fuel (e.g., H_2_) oxidation, where the gas-filled pore space meets both the ion conductor (YSZ) and electron conductor (Ni) phases:

(1) $$H_{2}\left(gas\right)+O^{2-}\left(YSZ\right)\to H_{2}O\left(gas\right)+2e^{-}(Ni)$$

**Figure 1:**
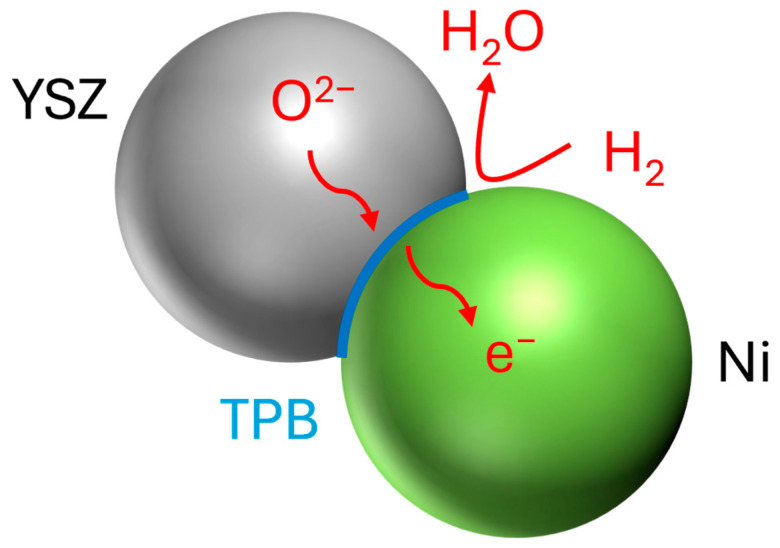
Schematics of hydrogen oxidation reaction occurring in Ni/YSZ cermet triple-phase boundary (TPB).

The junction of the three phases is known as the triple-phase boundary (TPB) (Figure 1). A higher TPB length offers more active sites and usually is in favor of the electrochemical reaction. In addition, the size of the reaction site is determined by the volume fraction, particle size, and arrangement of the metallic and ceramic phases. Hence, the accurate characterization and quantification of the TPB can help us better understand the performance of electrodes with different microstructures.

Unfortunately, it has been challenging to account for actual anode microstructures using both stereological analysis of SOFC images [20] and theoretical 3D models [21,22]. Wilson et al. [9] were the first ones to demonstrate the applicability of combined ion milling and SEM to the study of fuel cells. By combining FIB milling with SEM imaging, they were able to obtain a 3D model of the electrode microstructure with a voxel size smaller than 20 nm. They used the FIB to cut slices of a designated width through a certain layer of the cell. Then, they imaged each section using SEM and used software reconstruction to produce a 3D model of the fuel cell microstructure. The authors argued that digital modeling was essential for obtaining improved estimates of the reaction site length and represented the next reasonable step in heterogenous porous media quantification analysis.

Because 3D digital reconstruction enables a microstructural comparison between different electrodes, we believe it could provide an understanding of the effect of various cell fabrication parameters on SOFC performance. The fabrication of SOFC typically requires a high-temperature sintering process to achieve a gas-tight electrolyte. During sintering, densification of the composite powders occurs due to grain boundary diffusion, which leads to material relocation from the surface of the particle at the grain boundary to the particle necks. Because densification is attained through particle neck growth, the particle shape is not altered [23]. The sintering temperature is determined by the melting temperature of the materials used and has a direct impact on the anode microstructure [24,25]. An optimized sintering temperature should be high enough to densify the electrolyte layer while as low as possible to minimize anomalous grain growth. In this study, we use high-resolution FIB-SEM techniques to mill into the anode functional layer and image the exposed microstructures of two electrochemical cells prepared under different sintering temperatures. A comparison between the two cells was completed to determine differences in total and connected porosity, pore and particle size, triple-phase boundary (TPB) density, and hydrogen flow through the anode based on temperature variation during sintering of the electrolyte–fuel electrode bilayers. The high electrochemical performance is rationalized with the improved microstructure by lowering the sintering temperature.

## 2. Materials and Methods

### 2.1. SOFC Fabrication and Electrochemical Characterization

SOFC substrate tape was fabricated through the tape-casting process followed by the lamination process. The tape was cut and sintered at two different temperatures for 3 h to obtain an electrolyte–anode bilayer that consisted of a Y_0.16_Zr_0.84_O_1.92_ (YSZ) electrolyte (Tosoh Company, Tokyo, Japan), a YSZ/NiO functional layer, and a YSZ/NiO anode support layer. The bilayer substrates sintered at 1450 °C and 1365 °C are denoted as *T1* and *T2*, respectively. A Gd_0.2_Ce_0.8_O_1.9_ (Fuelcellmaterials, Lewis Center, OH, USA) interlayer was screen-printed on the YSZ electrolyte and sintered at 1200 °C for 2 h. La_0.6_Sr_0.4_Co_0.2_Fe_0_._8_O_3_ (Fuelcellmaterials, Lewis Center, OH, USA) was applied as the cathode with an area of 2 cm^2^ and sintered at 1080 °C for 30 min. The electrochemical performance of the cell was evaluated in fuel cell mode with 500 sccm air and 200 sccm humidified hydrogen. The cell voltage, as a function of current density, was collected at a scan rate of 5 mV/s by using a Biologic VMP-3 potentiostat (Franklin County, PA, USA). The electrochemical impedance spectra at open circuit voltage (OCV) were acquired from 0.1 Hz to 50 kHz with an AC amplitude of 10 mA/cm^2^. The quality of impedance was validified with the Kramers–Kronig test by Lin-KK Tools with a less than 1% error over the frequency range [26].

### 2.2. Focused Ion Beam–Scanning Electron Microscopy (FIB-SEM)

**Figure 2:**
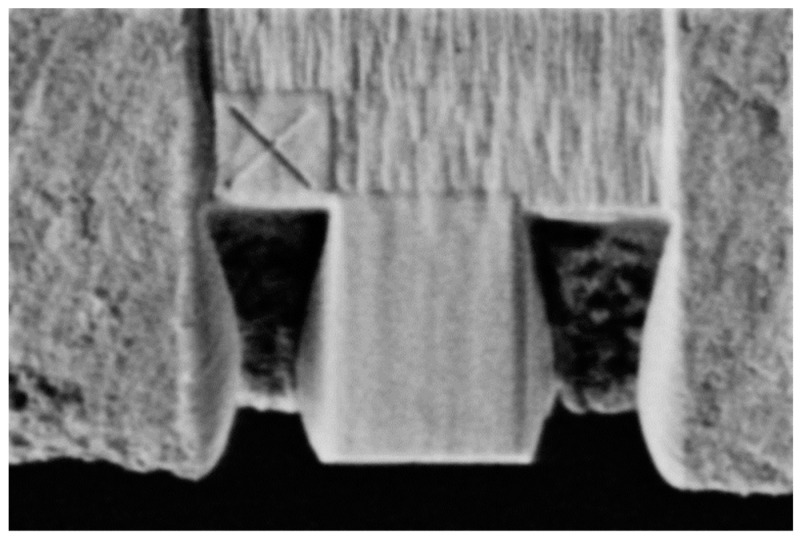
SEM image showing the area of interest delimited by the two trenches and the fiducial used for drift corrections.

Data were obtained using a Scios 2 Dual Beam focused ion beam (FIB) and scanning electron microscope (SEM). Both samples, *T1* and *T2*, were broken to provide a fracture face from the center of the fuel cell, and one of the obtained halves was mounted with carbon tape on a 52° pre-tilted holder. A 2 nm platinum surface protection layer was deposited over the area of interest to prevent charging, and a fiducial was added to aid in drift correction during milling and imaging. Trenches were created on the left and right sides of the area of interest to accommodate debris during edge milling (Figure 2).

The ThermoFisher Auto Slice and View 4.1 software (Waltham, MA, USA) was used for milling. The focused ion beam high voltage was set to 30 kV with a beam current of 5 nA. Rocking mill mode was set at a 5° tilt angle to reduce curtaining. In total, 485 slices 20 nm apart were imaged using an Everhart Thornley secondary electron (ETD-SE) detector (Waltham, MA, USA). Every second slice was captured with a resolution of 1536 × 1024 @ 8 bit and an acquisition rate of 5 microseconds, resulting in a total of 242 SEM images for the *T1* sample and 300 SEM images for the *T2* sample, with a voxel size of 23.8 × 23.8 nm. Energy dispersive spectroscopy (EDS) mapping was performed on the last slice of each sample at 20 kV to determine the elemental concentrations in the phases observed in the functional layer and to help recognize the three anode phases (nickel, yttrium-stabilized zirconia, and pores).

### 2.3. 3D Data Pre-Processing

The SEM images were imported into Avizo 2021.1 to extract volumes. In the pre-processing phase, bounding areas that were highly affected by charging artifacts that could affect phase segmentation results were cropped out. This resulted in two volumes of 15.34 μm × 18.65 μm × 8.26 μm (for *T1*) and 18.18 μm ×11.16 μm × 12.66 μm (for *T2*), which were further filtered to prepare the images for the segmentation step. A Fast-Fourier Transform (FFT) stripe filter (Waltham, MA, USA) with a tolerance of 3 was used to eliminate any remaining vertical curtaining. To denoise the images, a non-local means filter with a cubic search window of 10 pixels was applied, and the two volumes were resampled to cubic voxel size to ensure data compatibility with the porosity modules. A grayscale value histogram of the resampled, denoised SEM images was then used to assign a label to each pixel using a thresholding tool. Lower and upper bounds were selected on the histogram to differentiate the nickel, YSZ, and pore phases in the anode. Once phase segmentation was completed, the two samples were reconstructed in 3D to evaluate anode composition and microstructure.

### 2.4. Pore and Grain Network Extraction

It was previously shown that anode pore and grain size distributions, together with the Ni/YSZ ratio obtained after sintering, have a major impact on the electrode microstructure and function [27,28]. For anode pore network analysis, floating pores were removed. Connected porosity was preserved by retaining all regions labeled as porosity-present in two consecutive parallel planes, ensuring they shared at least one common vertex in the desired direction. The connected porosity was then separated into individual pores to allow for pore size variation analysis in the *x*-, *y*-, and *z*-directions. Pore separation served as a prerequisite for pore network modeling, which was used to approximate anode pore structure, reveal pore arrangement, pore–throat connectivity, and pore and pore–throat size distribution. In addition, skeleton modeling allowed for a comparison of individual pore radii. To separate the nickel and yttria-stabilized zirconia grains for statistical metrics, a watershed-based algorithm was used. The computed pore and grain radii corresponded to spheres of the same volume as the analyzed objects.

### 2.5. Triple-Phase Boundary (TPB) Identification and Quantification

To quantify the triple-phase boundary, only those locations where three anode phases met were selected. This was achieved by using individual segmentation labels to detect voxels that had at least one common vertex. Once an interface between the connected pores, nickel, and YSZ was identified, skeletonization was performed, and the TPB was displayed using spatial graph reconstruction. This allowed for a detailed analysis of the studied interface and quantification with respect to segment length. The total length of the electrochemical reaction site was obtained by summing up the length of all individual segments. The density was then calculated with respect to the sample’s physical volume:

(2) $$TPB\;density\left[\frac{1}{{\mu m}^{2}}\right]=\frac{TPB\;length\;[\mu m]}{Physical\;sample\;vol.[{\mu m}^{3}]}$$

The anode microstructure was recreated using Aviso 3D modeling, allowing us to detect the exact location of reaction sites and their extent. Hence, this technique eliminates the need for using a hypothetical microstructure where pores and grains are regarded as randomly packed spheres and, subsequently, offers an accurate estimate of the triple-phase boundary by identifying its extent with respect to pore and grain distribution.

### 2.6. Hydrogen Flow Simulation

The Pergeos 2021.1 software was used to simulate an absolute permeability experiment constrained by inlet and outlet pressures, where anode permeability was intrinsically dictated by porosity and pore size distribution. Hydrogen with a viscosity of 2 × 10^−5^ Pa·s was fed to the anodes of the two SOFCs. Hydrogen flow through the anode was simulated in the y-direction, namely, from the bottom of the anode to its top. To perform the simulation, inlet pressure was considered to be 101,325 Pa, and outlet pressure at the top of the anode–electrolyte boundary was set to 101,315 Pa. Darcy’s law was employed to calculate anode permeability for a given gas viscosity.

## 3. Results and Discussion

### 3.1. Anode Phase Reconstruction and Quantification

**Figure 3:**
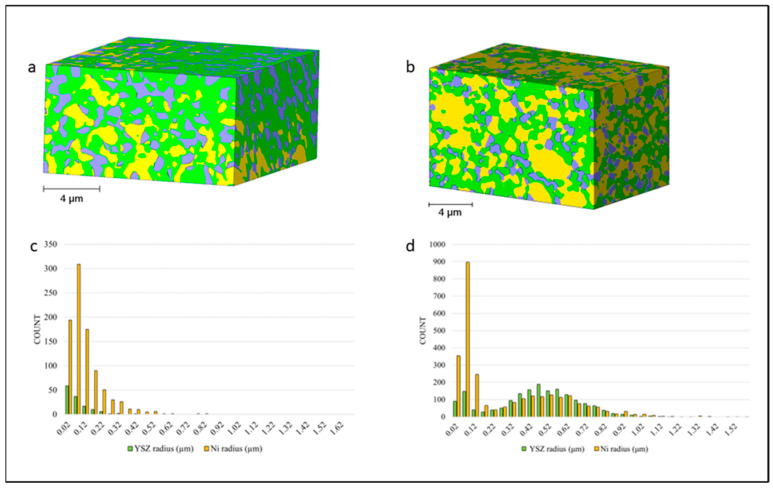
A 3D reconstruction showing the three phases of the anode of (**a**) cell *T1* and (**b**) cell *T2*. (**c**,**d**) show corresponding grain size distribution using histograms. YSZ is shown in green, nickel in yellow, and the pore phase in purple.

The volume percentage and characteristic size of each component of *T1* and *T2* in the *x*-, *y*-, and *z*-directions are presented in Table 1 and Table 2. Sample *T1* has 2.4 times more yttria-stabilized zirconia (55.44 vol.%) than nickel (22.86 vol.%), with a total porosity of 21.70 vol.%. The connected pore space accounts for 16.38 vol.% of the total volume. The 3D representation of the sample sintered at a higher temperature (Figure 3a) supports the values obtained and shows that the volume is dominated by the YSZ phase. In comparison, the *T2* anode, sintered at a lower temperature (Figure 3b), has about equal amounts of YSZ (41.21 vol.%) and nickel (44.95 vol.%) and lower total porosity (14.91 vol.%) and connected pore space (10.65 vol.%).

Volume segmentation revealed that nickel particles had clear boundaries and were easily distinguishable, but the ceramic phase was closely compacted and comprised fused grains. The metallic phase of the anode was porous and easily observed (Figure 3a,b). Table 1 shows the YSZ grains and nickel particles to be the same size. Table 2 shows that the YSZ grains were larger than the nickel particles, which agrees with observations made by Chen et al. [29]. The authors explained that smaller nickel grains are better because they can contribute to mitigating the thermal expansion mismatch. Grain size distributions in Figure 3c,d appear to be skewed, which indicates that the standard deviation cannot be treated as a measure of data spread in both directions. Sample *T1* was characterized by grain radius distributions with long right tails, which is caused by a population of relatively large grains. In this case, the standard deviation can be used to assess the spread of the data in the right direction. While the mean of the YSZ grain size does not exceed 0.24 μm in sample *T1*, the anode of cell *T2* is characterized by metallic and ceramic phases with larger particles. The YSZ phase has a mean grain radius of 0.42 μm.

**Figure 4:**
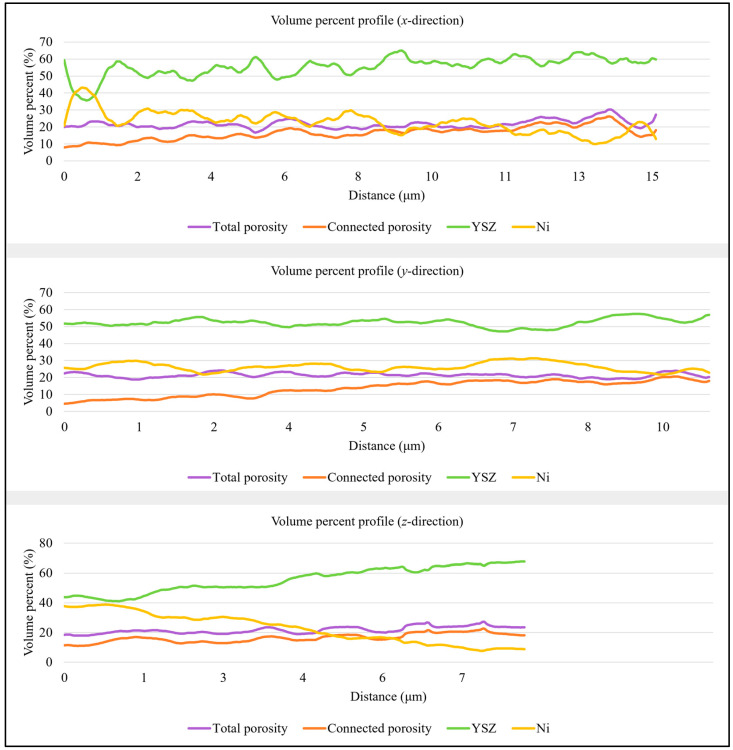
From top to bottom: anode phase volume percent variation profiles in the *x*-, *y*-, and *z*-directions obtained for cell *T1*. Total porosity is shown in purple, connected porosity in orange, YSZ in green, and nickel in yellow.

**Figure 5:**
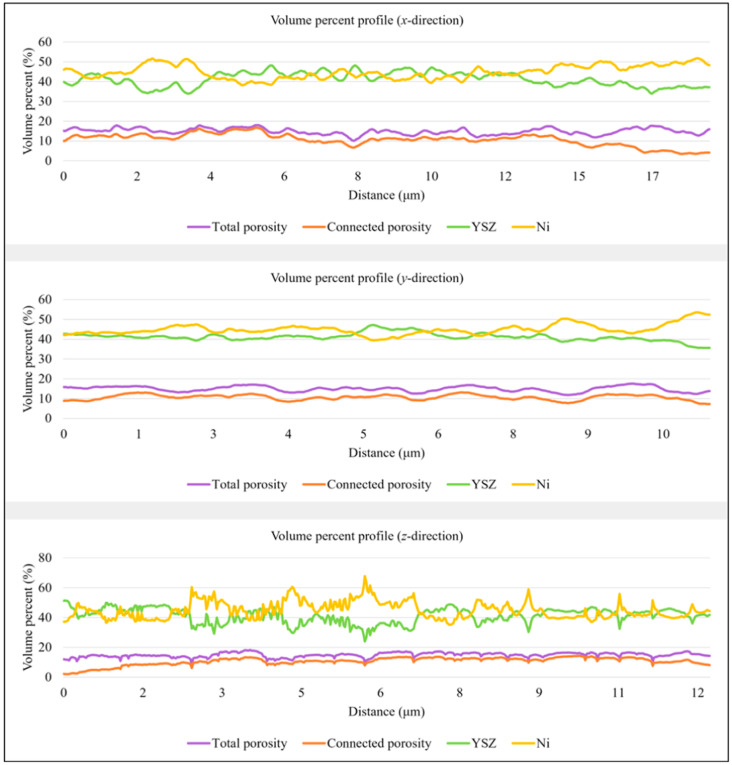
From top to bottom: anode phase volume percent variation profiles in the *x*-, *y*-, and *z*-directions obtained for cell *T2*. Total porosity is shown in purple, connected porosity in orange, YSZ in green, and nickel in yellow.

Phase variation across the two volumes was studied in Figure 4 and Figure 5, providing insights into the dependencies between the three phases. The three profiles can be interpreted as follows: The profile in the *x*-direction shows volume percent variation parallel to the anode–electrolyte boundary, while the second profile presents phase volume changes from the inlet to the outlet of the anode. To investigate compositional changes across the thickness of the anode, the extent of which is determined by the number of slices milled through the sample, a third profile is generated along the *z*-direction within the volume. In all graphs, the *x*-axis represents the distance parallel to the direction of investigation from the system’s origin to the analyzed location. The two profiles in the y-direction exhibit different behaviors. In sample *T1*, there is a very weak negative relationship between nickel and connected porosity, with a correlation coefficient of only −0.02, making their relationship not practically significant. In contrast, in sample *T2*, a correlation coefficient of −0.61 describes the relationship between nickel and total porosity volumes, with pore space increasing where less nickel is present. This is a direct consequence of nickel particles being replaced by pore space. In both samples, nickel and porosity volumes are inversely proportional in all directions. According to the statistical analysis in Table 1 and Table 2, both the ceramic and metallic phases show greater variation across the anode thickness, while connected porosity exhibits more significant variations from the inlet to the outlet of the anode in sample *T1*, with a standard deviation of 4.74 vol.%. Conversely, sample *T2* displays more variation in the connected pore space parallel to the anode–electrolyte boundary, with a standard deviation of 3.08 vol.%.

For a comparative 2D analysis, samples were obtained from five different regions from each sample to investigate porosity volume. A mean of 19.00 vol.% with a standard deviation of 1.40 vol.% was obtained for *T1*, and a mean of 13.18 vol.% and a standard deviation of 1.27 vol.% was obtained for *T2*. The volumes and standard deviations had values similar to the ones obtained for the 3D analysis of *T1* and *T2* (Table 1 and Table 2). This appears to be a relatively quick and accurate way to obtain limited data without the time and expense of milling. It also shows that the area selected for 3D analysis is representative of other areas in the sample.

### 3.2. Connected Pore Space Modeling for TPB Characterization

**Figure 6:**
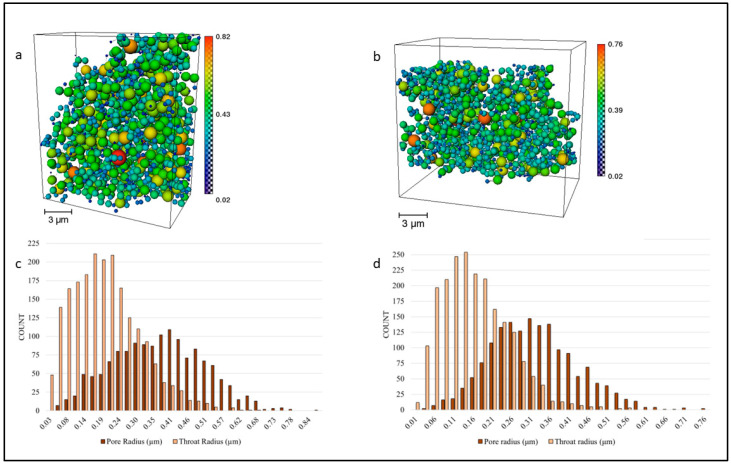
Connected pore network representation using estimated pore size for (**a**) cell *T1* and (**b**) cell *T2*. Pore radius scales are displayed beneath the images. (**c**,**d**) show pore size and pore throat size distribution using histograms for *T1* and *T2*, respectively. Orange is used for the pores and light orange for the throats of the network.

One prerequisite of an active electrochemical reaction site is for the porous space to be connected to allow for uninterrupted hydrogen flow from the inlet to the outlet of the anode. The connected pore space for *T1* and *T2* was isolated from the initial total porosity, with any remaining unconnected pores identified as floating pores. The resulting structure was then examined. For both the *T1* (Figure 6a) and *T2* (Figure 6b) samples, the connected pore space is continuous across the *y*-direction, enabling pore characterization without the need for further pre-processing. The obtained pore network model (Figure 6) shows that the *T1* network has pores with radii between 0.02 and 0.82 μm with a mean of 0.35 μm and the pore throats are almost two times smaller than the pores. The *T1* distributions are displayed in Figure 6c. The *T2* pore network pertaining to the sample sintered at a lower temperature is characterized by a normally distributed pore space up to 1.52 μm in diameter and a mean of 0.31 μm (Figure 6d). However, interestingly, as is the case for *T1*, the throats of the *T2* network are two times smaller than the pores with a mean throat size of 0.15 μm.

**Figure 7:**
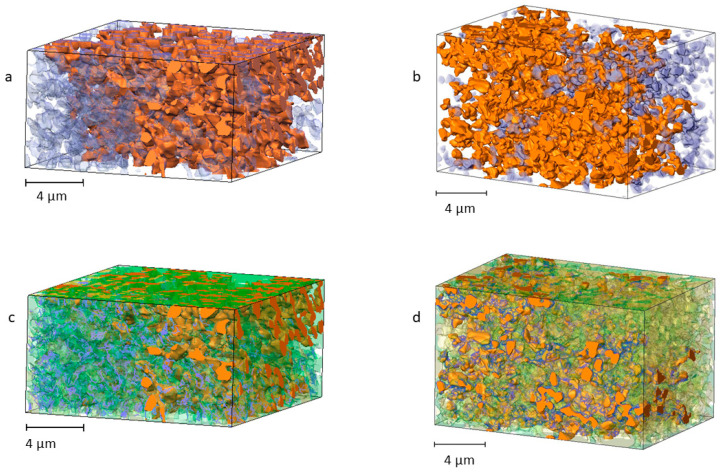
3D reconstruction of the connected pores (in orange) and floating pore space (in purple) for (**a**) cell *T1* and (**b**) cell *T2*. The corresponding triple-phase boundaries (in blue) are displayed on top of the connected pore space in images (**c**) and (**d**), respectively.

The triple-phase boundary comprises all active reaction sites where the connected pore space meets with the metallic and ceramic phases of the anode. The connected porosity was only used to identify such sites and represent their length using individual segments. The TPB is displayed in Figure 7c,d and encompasses all triple junctions where the boundaries of the three phases meet. The lengths of the separate segments were summed up to obtain the total length of the reaction site in each sample (Table 3). For the ROI corresponding to cell *T1*, the TPB has a length of 4618.99 μm, which results in a density of 1.96 μm^−2^ for a total sample volume of 2363.11 μm^3^. In contrast, sample *T2* is characterized by a much higher TPB density of 3.92 μm^−2^, corresponding to a sample volume of 2568.59 μm^3^. The higher TPB density of the second sample is the result of a more extensive pore network and a balanced Ni/YSZ volume ratio. The increased sintering temperature used for T1 apparently led to the formation of smaller nickel particles unable to support porosity formation, which subsequently led to a decrease in the electrochemical reaction site.

### 3.3. Anode Permeability and Cell Performance

**Figure 8:**
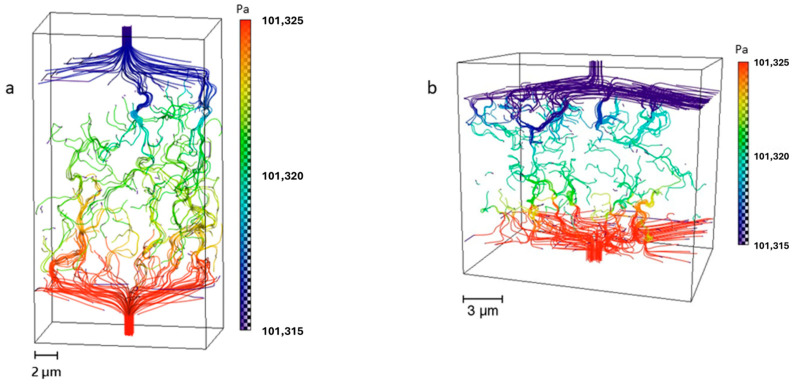
Pressure field evolution inside the connected network of the two anode samples. Both (**a**) cell *T1* and (**b**) cell *T2* exhibit good pore connectivity, which allows for gas flow from the inlets to the outlets of the respective studied volumes.

Hydrogen flow simulations were performed for regions corresponding to the connected pore space. Both samples, *T1* and *T2*, exhibit similar pore networks, leading to comparable hydrogen flow behavior. The pressure field evolution from the inlet (higher pressure) to the outlet (lower pressure) within the investigated anode regions is depicted in Figure 8a,b for samples *T1* and *T2*, respectively. The permeability obtained for the anode volume of sample *T1* is *k* = 8.8 × 10^−3^ md, while *T2* has a slightly lower anode permeability of *k* = 8.0 × 10^−3^ md. Electrode permeability can provide additional insights into the performance of the two cells. Moreover, Geagea et al. [30] showed that high permeabilities are linked to durable gas pathways, which effectively mitigate diffusional polarization losses.

**Figure 9:**
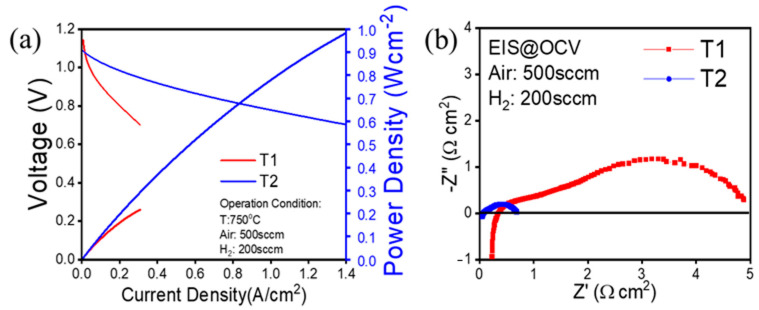
(**a**) Current density–voltage–power density relationship of two SOFCs (*T1* and *T2*) sintered at different temperatures. (**b**) Electrochemical impedance spectra (EIS) at an open circuit of two SOFCs (*T1* and *T2*) sintered at different temperatures.

Figure 9a shows the current density–voltage–power density (i-V-P) relationship of the two cells. Cell *T1* is characterized by low power density at 0.75 V (0.19 W/cm^2^), whereas cell *T2* has a power density of 0.86 W/cm^2^. Both cells have high open circuit voltages (>1.09 V), which indicate a densified electrolyte and a good seal. Electrochemical impedance spectra (EIS) (Figure 8b) show that cell *T1* has higher total cell resistance (4.88 Ω·cm^2^) than cell T2 (0.69 Ω·cm^2^), which is generally contributed by the polarization resistance of the electrodes. These characteristics are a result of the anode microstructure obtained using different sintering temperatures. The phase arrangement in sample *T1* was significantly affected by the relatively high sintering temperature, resulting in a densely packed mass of yttria-stabilized zirconia with limited nickel dispersion. This lack of effective interaction between the phases adversely impacts power density and hinders the electrochemical reaction, even when there is interconnected pore space. Consequently, this manifests as a pronounced low-frequency arch in the impedance spectrum, signifying high diffusion impedance. Moreover, the relatively short triple-phase boundary (TPB) length in sample *T1* leads to sluggish kinetics across the entire functional layer, further exacerbating the polarization impedance. In contrast, the superior performance of cell *T2* is corroborated by the presence of a well-defined and extensive pore network, along with an extended TPB length, as clearly demonstrated in the FIB-SEM reconstruction.

## 4. Conclusions

Focused ion beam (FIB)–scanning electron microscopy (SEM) allowed for the characterization of the microstructure of two solid oxide fuel cells prepared at different sintering temperatures. A 3D volume reconstruction showed that a relatively low sintering temperature significantly and positively affected the distribution, volume, and particle size of yttria-stabilized zirconia, nickel, and pore phases inside the anode, as well as the extent of the important triple-phase boundary interface. The poor performance of the *T1* sample, sintered at a higher temperature, is explained by the poorly connected pore network and very low-density triple-phase boundary. The phase distribution within the *T1* anode fails to provide a continuous electrochemical reaction site of reliable density in the absence of nickel, even though the connected pore space system exhibits favorable gas permeability. In contrast, the *T2* sample, sintered at a lower temperature, had approximately equal amounts of YSZ and nickel and larger pores, which enabled the formation of significantly more TPB electrochemical reaction sites. The higher power density of the *T2* cell was also the result of its robust pore network, capable of transporting hydrogen throughout the anode. The methodology used in this paper eliminates the need to employ hypothetical structures and provides accurate estimates of the investigated parameters by evaluating microstructures that were successfully reconstructed using high-resolution microscopy techniques.

## Figures and Tables

**Table 1:** Volume percent and size of the YSZ, nickel, and total and connected porosity phases in sample *T1*. The size of the pores was quantified using the connected pore network, and thus, no values are shown for the total porosity.

***T1***	**Anode Phase**	**Volume Percent (vol.%)**				**Size (μm)**	
		**Mean**	**STD** ***x*-Direction**	**STD** ***y*-Direction**	**STD** ***z*-Direction**	**Mean**	**STD**
	YSZ	55.44	5.67	4.33	8.35	0.24	0.73
	Ni	22.86	6.40	4.54	10.03	0.24	0.27
	Total porosity	21.70	2.44	1.66	2.27	-	-
	Connected porosity	16.38	3.94	4.74	2.87	0.35	0.14

**Table 2:** Volume percent mean and variation in the *x*-, *y*-, and *z*-directions along with particle size in sample *T2*. The connected pores were used to quantify pore size.

***T2***	**Anode Phase**	**Volume Percent (vol.%)**				**Size (μm)**	
		**Mean**	**STD** ***x*-Direction**	**STD** ***y*-Direction**	**STD** ***z*-Direction**	**Mean**	**STD**
	YSZ	41.21	3.53	2.01	4.92	0.42	0.23
	Ni	44.95	3.43	2.68	5.61	0.26	0.28
	Total porosity	14.91	1.56	1.40	1.55	-	-
	Connected porosity	10.65	3.08	1.36	2.64	0.31	0.11

**Table 3:** Triple-phase boundary parameters for the studied cells.

	*T1*	*T2*
Sample volume (μm^3^)	2363.11	2568.59
TPB length (μm)	4618.99	10,062.40
TPB density (μm^−2^)	1.96	3.92

## Data Availability

Data are contained within the article.
